# DSM-5 Criteria for Alcohol and Cannabis Use Disorders: Are Older Adults Less Likely to Endorse Certain Criteria?

**DOI:** 10.3390/ijerph22060843

**Published:** 2025-05-28

**Authors:** Namkee G. Choi, Jeffrey A. Morris, C. Nathan Marti

**Affiliations:** Steve Hicks School of Social Work, University of Texas at Austin, 405 W 25th Street, Austin, TX 78705, USA; jeffreymorris@utexas.edu (J.A.M.); nate.marti@utexas.edu (C.N.M.)

**Keywords:** DSM-5, older adults, alcohol use disorder, cannabis use disorder, social impairment, physical and psychological problems

## Abstract

With increasing substance misuse among older adults, we examined the question of whether older adults may be less likely to endorse certain DSM-5 criteria for alcohol and cannabis use disorders (AUD and CUD). We used the 2021–2023 National Surveys on Drug Use and Health (*N* = 17,494 for AUD and *N* = 12,264 for CUD) and descriptive statistics to compare the 65+ and under 65 age groups in their endorsements of 11 DSM-5 criteria. A multivariable logistic regression model was fitted for each criterion as the dependent variable with the age group as the independent variable and other characteristics as covariates. For AUD, the 65+ age group was associated with lower odds of endorsing seven out of eleven DSM-5 criteria, including social impairments (e.g., failure to fulfill role obligations (aOR = 0.30, 95% CI = 0.16–0.56); social problems (aOR = 0.46, 95% CI = 0.30–0.71); given-up activities (aOR = 0.66, 95% CI = 0.47–0.94); hazardous use (aOR = 0.53, 95% Yes CI = 0.34–0.81); and physical/psychological problems (aOR = 0.51, 95% CI = 0.37–0.70). For CUD, the 65+ age group was less likely than the under 65 age group to endorse hazardous use (aOR = 0.04, 95% CI = 0.01–0.17) and withdrawal (aOR = 0.39, 95% CI = 0.20–0.73 for criterion A and aOR = 0.16, 95% CI = 0.05–0.48 for criterion B). Clinicians should be aware that older adults might not express the full range of symptoms in the same way as the younger age groups. A more nuanced understanding of older adults’ social context may be needed for accurate diagnosis.

## 1. Introduction

The prevalence of substance use disorder (SUD) among older adults (ages 65 and older) has increased over the past two decades. This increase has been attributed to longer life expectancy, the aging of the baby boom generation that has had more liberal attitudes toward substance use, increased prescription medication use, and the expanded legalization and increasingly permissive attitudes toward cannabis and its derivatives, along with their increased availability and marketing/advertising [1,2,3,4,5,6]. Older adults are more vulnerable to the adverse effects of substance use and misuse, as it can worsen chronic illnesses, functional impairments (e.g., from substance-affected falls and fall injuries), and psychological and cognitive health problems [6]. Aging-related changes in pharmacokinetics and pharmacodynamics and the interactions with prescription drugs can result in serious adverse outcomes in the cardiovascular, digestive, endocrine, and central nervous systems [7,8,9,10].

Given SUD’s adverse physical, psychological, and cognitive effects in later life, it is essential to screen for and identify substance misuse and SUD in older adults, as screening and diagnosis are the first steps toward treatment engagement. Research has shown notable increases in publicly funded SUD treatment admissions among those ages 55 and older over the past two decades [11,12,13,14]. These studies indicated that while an increasing share of these admissions included substances other than alcohol, alcohol use disorder (AUD) remained the most common primary SUD in this age group, accounting for over 60% of all admissions. Treatment admission data also showed that nearly one in six cases aged 55 and older involved cannabis or cannabis use disorder (CUD) [15]. Older adults have been the fastest-growing group of cannabis users, with 8.0% of the 65+ age group in 2022 reporting past-year cannabis use, compared to 2.9% in 2015–2016 [16,17]. Despite the increases in older adult SUD treatment admissions, the 2022–2023 National Survey on Drug Use and Health (NSDUH) showed that less than one in seven adults with SUD overall, and even lower proportions of older adults, reported receiving SUD treatment in the past year [18]. Furthermore, although older adults have more contact with healthcare providers than younger adults, most older adult treatment admissions were not referred by healthcare providers, suggesting room for improvement in SUD screening and referral to treatment [6].

The symptom checklists/criteria in the fifth edition of the Diagnostic and Statistical Manual (DSM-5) are the gold standard for diagnosing psychiatric and substance use disorders [19]. Studies have shown that the DSM-5 SUD formulation tends to be substantially more inclusive than the DSM-IV formulation, leading to the identification of a larger number of mild-severity individuals (who meet 2–3 diagnostic criteria out of 11) being captured [20,21]. For example, Bailey et al. [21] showed the estimated past-year DSM-5 AUD prevalence rate in the 2020 NSDUH was 10.1%, compared with a 5.4% rate of past-year DSM-IV alcohol abuse or dependence, without increases in drinking behavior. Choi et al.’s study [16] based on the 2022 NSDUH also reported an increase in the DSM-5 past-year CUD rates in the adult population to 7.0% in 2022 from 6.3% in 2017 per Compton et al. [22], with the increase exclusively due to the rise in mild CUD. However, unlike the case of AUD, the increase in CUD likely also reflected the substantial increase in cannabis use and use frequency. Caulkins [23] showed that between 1992 and 2022, daily/near-daily cannabis users aged 12 and older grew from 0.7 million to 17.7 million, which surpassed the number of alcohol users. Especially, with the legalization of medical cannabis use in the majority of the U.S. states, older adult medical cannabis users who tend to use it more frequently than nonmedical users have been increasing [24].

While the DSM-5 SUD symptom checklists may be more inclusive in identifying mild SUD, research has also suggested that some checklist items may be subject to age-related biases, although other items, such as loss of control, craving, tolerance, and withdrawal, may apply to all age groups [25,26,27,28]. Specifically, older adults who are disengaged from the labor force, live alone or have limited social circles, drive less, and use substances at home in isolation from others are less likely to endorse social impairment items/criteria (i.e., failure to fulfill role obligations, giving up important social, occupational or recreational activities, and social problems) and hazardous use. A Canadian survey of older past-month cannabis users showed that they reported significantly higher loneliness than their peers who never used it [29], suggesting that many older cannabis users may have smaller social networks, fewer social interactions, and lower relationship conflicts. Age-related changes in role functioning and social circumstances may lead to the under-identification of SUDs, especially severe SUD cases among older adults. Mannes et al. [28] found that greater role/social functioning impairment resulted in greater odds of severe AUD (meeting 6–11 criteria). Another study of multinational samples of adults also found that social and interpersonal problems were the strongest indicators of DSM-5 severe AUD [30]. At the same time, older adults may also be less likely to endorse continued use despite the physical and psychological effects of substance use and hazardous use, which are also strongly associated with severe AUD [28]. Older adults may attribute physical and psychological problems to aging, not to substance misuse. Research has also shown that older adults are most likely to reduce alcohol consumption based on healthcare professional advice, tangible adverse health effects, and potential interactions with medications [31,32,33]. A qualitative study of boomer cannabis users also showed that these individuals used cannabis in ways that minimized physical and social harm [34].

Despite the likely age group differences in endorsing diagnostic criteria and their significant implications for identifying SUDs in older adults, little empirical research has examined the extent of such differences. The primary aim of this exploratory/descriptive study, based on nationally representative epidemiologic data on U.S. adults (ages 18+) with AUD or CUD, was to compare the 65+ age group with the under 65 (18–64 years) age group regarding their endorsement of the 11 DSM-5 SUD symptom criteria. The study hypotheses were (H1) compared to the under 65 age group, the 65+ age group would have lower odds of endorsing criteria for social impairments (failure to fulfill role obligations; social, occupational, or recreational activities given up; continued use despite social problems), continued use despite physical and psychological problems, and recurrent use in physically hazardous situations and (H2) no significant associations would exist between age group and the other DSM-5 criteria, controlling for other demographic characteristics and self-rated health. The findings will contribute to a better understanding of potential age group differences in the DSM-5 symptoms of AUD and CUD and of the need for age-appropriate SUD assessments for older adults.

## 2. Materials and Methods

### 2.1. Data Source

Data for this study came from the 2021–2023 NSDUH, funded by the U.S. Substance Abuse and Mental Health Services Administration. The NSDUH is an annual epidemiologic survey of the civilian, non-institutionalized population, aged 12 years and older, to measure the prevalence of substance use, mental illnesses, SUDs, behavioral health treatment, physical/functional health, and healthcare utilization. The NSDUH began using DSM-5 criteria for SUDs in 2021, following a pilot implementation of DSM-5 alongside DSM-IV in its 2020 survey. The NSDUH uses a multi-stage, stratified sampling design, with states as the first level of stratification, and an independent, multi-stage area probability sample within each state and the District of Columbia [35]. Because of the COVID-19 pandemic, data in 2021–2023 were collected using both in-person and web-based modes. The overall percentages of interviews that were completed via the web were 54.6% in 2021, 40.7% in 2022, and 36.0% in 2023 [36]. The combined number of adult respondents in the 2021–2023 NSDUH was 139,524 (47,291 in 2021, 47,100 in 2022, and 45,133 in 2023). The analytic samples in this study were those who were diagnosed with past-year AUD (*N* = 17,494) or CUD (*N* = 12,264).

### 2.2. Measures

#### 2.2.1. DSM-5 SUD Criteria

The criteria include the following 11 items: (1) taking the substance in larger amounts or for a longer duration than intended; (2) unsuccessful efforts to cut down or control use (loss of control); (3) spending a great deal of time obtaining, using, or recovering from use of the substance; (4) intense cravings or a strong urge to use; (5) recurrent use despite failing to fulfill major role obligations at work, school, or home; (6) continued use despite social problems; (7) giving up or reducing important social, occupational, or recreational activities (given-up activities); (8) recurrent use in physically hazardous/dangerous situations; (9) continued use despite a physical or psychological problem that could have been caused or made worse by the substance; (10) an indication of increased tolerance, i.e., needed more of the substance to obtain the same effect; and (11) the development of withdrawal symptoms (a), which can be relieved by taking more of the substance (b). For past-year AUD and CUD diagnoses, all NSDUH respondents were queried about these 11 criteria (yes = 1, no = 0 for each) if they used alcohol and cannabis, respectively, on six or more days in the preceding 12 months. Mild, moderate, and severe symptom severities are characterized by meeting 2–3, 4–5, and 6–11 criteria.

#### 2.2.2. Age Groups and Covariates

We followed the NSDUH categories (18–25 years, 26–34 years, 35–49 years, 50–64 years, and 65+ years), and then the 18–64 age groups were combined as the under 65 age group. Covariates in this study were gender (male, female); race/ethnicity (non-Hispanic White, non-Hispanic Black, Hispanic, non-Hispanic Asian and pacific islander, American Indian/Alaska Native, multi-racial); education (<high school, some college/associate degree, college degree); work status (employed full time, employed part time, unemployed, other); and self-rated health (on a 5-point scale: 1 = poor through 5 = excellent).

### 2.3. Analysis

We used Stata/MP 18’s svy function (College Station, TX, USA) with the subpop command in all analyses to account for NSDUH’s multi-stage, stratified sampling design to ensure that variance estimates incorporate the full sampling design. For this study’s 3-year pooled data set, adjusted person-level analysis weights were created by dividing the final person-level analysis weights by the number of years of combined data (three in the current study) following NSDUH guidelines for combining sampling weights across multiple survey years. All estimates presented in this study are weighted, except sample sizes. First, we used Pearson’s χ^2^ tests to examine the differences in the prevalence of past-month and past-year alcohol and cannabis use and past-year AUD and CUD in the five age groups (18–25, 26–34, 35–49, 50–64, and 65+). Second, focusing on those with past-year AUD or CUD, we used Pearson’s χ^2^ tests and *t*-tests to compare gender, race/ethnicity, education, work status, and self-rated health between the 65+ and under 65 age groups. Third, we used Pearson’s χ^2^ tests to compare the age group differences in the percentages of those with AUD and CUD, respectively, who endorsed each of the 11 symptom criteria. We used *t*-tests to compare the total number of endorsed criteria and used Pearson’s χ^2^ tests to compare the symptom severity (mild, moderate, and severe) distributions. Fourth, to test the study hypothesis (associations between age group and SUD criteria), we fitted 12 separate logistic regression models per AUD and CUD, with each DSM-5 criterion as the dependent variable, age group (65+ vs. under 65) as the independent variable, and other demographic variables and self-rated health as covariates. (We used two separate models for the two [A and B] withdrawal criteria.) For sensitivity analyses, we also fitted logistic regression models per AUD and CUD for each SUD criterion with the five age groups, with the 35–49 age group as the reference category, as the independent variable. Because the sensitivity analysis results of both AUD and CUD pertaining to the 65+ age group did not deviate from those of the 65+ age group vs. the under 65 age group comparison, they are not reported. We present the logistic regression results as adjusted odds ratios (aORs) with 95% confidence intervals (CIs). As a preliminary diagnostic, the variance inflation factor (VIF), using a cut-off of 2.50 [37], from linear regression models was used to assess multicollinearity among covariates. VIF diagnostics indicated that multicollinearity was not a concern. Significance was set at *p* < 0.05.

## 3. Results

### 3.1. Prevalence of Alcohol and Cannabis Use and Use Disorder, 2021–2023, by Age Group

Table 1 shows that of the U.S. adult population, 67.1% and 22.0% used alcohol and cannabis, respectively, in the past year, and 11.2% and 6.6% had respective disorders. The 65+ age group had the lowest rates of AUD (4.8%) and CUD (0.9%), and the 18–25 and 26–34 age groups had the highest rates (15.4% for AUD and 15.9% for CUD in the 18–25 age group; 16.7% for AUD and 11.7% for CUD in the 26–34 age group), followed by the 35–49 and 50–64 age groups.

### 3.2. Demographic and Health Characteristics of Individuals with AUD or CUD by Age Group

AUD: Table 2 shows that of those with past-year AUD, 9.5% were in the 65+ age group. Compared to the under 65 age group, the 65+ age group included a higher proportion of non-Hispanic Whites but a lower proportion of full-time workers; however, the two age groups did not differ in terms of gender and education. The 65+ age group reported better self-rated health.

CUD: Table 2 shows that of those with past-year CUD, 3.1% were in the 65+ age group. Compared to the under 65 age group, the 65+ age group included fewer employed people; however, the two age groups did not differ in gender, racial/ethnic composition, and education. The 65+ age group reported better self-rated health.

### 3.3. Endorsement of SUD Criteria by Age Group

AUD: Table 3 shows that for both the 65+ and the under 65 age groups, craving was most frequently endorsed (66.4% of the 65+ age group and 69.1% of the under 65 age group), followed by larger amount/longer duration (56.8% and 66.1%), tolerance (45.3% and 51.1%), withdrawal (43.0% and 45.2%), time spent (39.9% and 69.1%), loss of control (38.9% and 33.6%), physical and psychological problems (19.0% and 31.0%), given-up activities (11.7% and 15.1%), social problems (9.3% and 16.8%), hazardous use (8.7% and 13.2%), and failure to fulfill role obligations (3.3% and 8.6%). Age group differences were statistically significant for the larger amount/longer criterion, time spent, physical and psychological problems, social problems, hazardous use, and failure to fulfill role obligations, with smaller proportions of the 65+ age group endorsing all these criteria. The 65+ age group endorsed an average of 3.4 criteria, and the under 65 age group endorsed 4.1 criteria (t = −5.39, *p* < 0.001). Of AUD symptom severity, 69.0%, 16.4%, and 14.6% of the 65+ age group and 53.8%, 24.3%, and 22.0% of the under 65 age group had mild, moderate, and severe, respectively, severities (overall age group difference: F[1.97,98.40] = 13.47, *p* < 0.001).

CUD: Table 3 shows that for both the 65+ and the under 65 age groups, time spent (79.4% of the 65+ age group and 81.0% of the under 65 age group) was most commonly endorsed, followed by craving (68.5% and 78.8%), tolerance (45.3% and 51.1%), withdrawal (41.6% and 65.6%), larger amount/longer duration (31.1% and 45.4%), loss of control (18.1% and 26.1%), physical and psychological problems (12.1% and 11.9%), given-up activities (7.7% and 14.0%), social problems (4.9% and 6.3%), failure to fulfill role obligations (3.6% and 6.6%), and hazardous use (0.2% and 3.5%). Age group differences were statistically significant for hazardous use and withdrawal only, with lower proportions of the 65+ age group endorsing these two criteria. The 65+ age group endorsed an average of 3.2 criteria, and the under 65 age group endorsed 4.0 criteria (t = −5.07, *p* < 0.001). Of CUD symptom severity, 70.6%, 25.4%, and 4.0% of the 65+ age group and 49.9%, 31.7%, and 18.3% of the under 65 age group had mild, moderate, and severe, respectively, severities (overall age group difference: F[1.61,81.00] = 5.17, *p* = 0.012).

### 3.4. Associations Between Age Group and Criterion Endorsement: Logistic Regression Results

In Table 4, each row of the first column shows each DSM-5 criterion as the dependent variable. Each row of the second (AUD) and fourth (CUD) columns shows the odds ratios for the 65+ age group versus the under 65 age group, adjusting for other demographic variables and self-rated health. Each row of the third (AUD) and the fifth (CUD) columns shows model statistics for each logistic regression model.

AUD: The second column in Table 4 shows that the 65+ age group was less likely than the under 65 age group to endorse larger amount/longer duration (aOR = 0.71, 95% CI = 0.52–0.96), time spent (aOR = 0.59, 95% CI = 0.44–0.78), failure to fulfill role obligations (aOR = 0.30, 95% CI = 0.16–0.56), social problems (aOR = 0.46, 95% CI = 0.30–0.71), given-up activities (aOR = 0.66, 95% CI = 0.47–0.94), hazardous use (aOR = 0.53, 95% CI = 0.34–0.81), and physical/psychological problems (aOR = 0.51, 95% CI = 0.37–0.70).

CUD: The fourth column in Table 4 shows that the 65+ age group was less likely than the under 65 age group to endorse hazardous use (aOR = 0.04, 95% CI = 0.01–0.17) and withdrawal (aOR = 0.39, 95% CI = 0.20–0.73 for criterion A and aOR = 0.16, 95% CI = 0.05–0.48 for criterion B).

## 4. Discussion

This study used the recent three years of nationally representative epidemiologic data on adults with AUD and CUD, respectively, to describe age-related patterns in endorsing DSM-5 SUD criteria. Comparing the 65+ and under 65 age groups is crucial for conducting age-appropriate clinical assessments of SUDs, which can, in turn, aid in enhancing treatment engagement among older adults with SUDs. Our findings indicate that the constellations of the most frequently endorsed criteria for AUD and CUD (i.e., by one-third to three-fourths of those with respective SUD) were identical for both age groups: craving, larger amount/longer duration, tolerance, withdrawal, and time spent. The next most frequently endorsed criteria were continued use despite physical/psychological problems (almost 1/3 of the under 65 age group and nearly 1/5 of the 65+ age group with AUD; 12% of both age groups with CUD) and given-up or reduced activities (15% of the under 65 age group and 12% of the 65+ age group with AUD; 14% of the under 65 age group and 8% of the 65+ age group with CUD). For those with AUD, the continued use despite social problems criterion was also endorsed by 17% of the under 65 age group and 9% of the 65+ age group.

### 4.1. AUD

Multivariable analyses showed that the 65+ age group had significantly lower odds of endorsing seven criteria out of eleven (i.e., except for loss of control, craving, tolerance, and withdrawal) for AUD. Compared to the under 65 age group, the odds of the 65+ age group endorsing failure to fulfill role obligations were less than one-third, and the odds of endorsing social problems, hazardous use, and continued use despite physical/psychological problems were approximately one-half. These findings support the study hypothesis that some DSM-5 criteria may not apply to certain older adults. As discussed, older adults are likely to face fewer external pressures to reduce drinking and less noticeable disruption in social roles or functioning, as they may have fewer social interactions to be affected by alcohol misuse. In their study of DSM-IV AUD criteria using item response theory analyses, Kuerbis et al. [38] also noted that reduced interactions with wider networks among older adults may result in more intense disturbances to smaller networks but endorsed only at a higher severity level. Some older adults might also have adjusted their drinking behavior to avoid social conflicts and for fear of stigma [39]. Older adults may also underreport or fail to recognize physical and psychological problems related to alcohol use. Some may attribute physical, mental, and cognitive health problems to aging itself, rather than alcohol consumption, and may even mistakenly believe that alcohol helps manage the related illness symptoms [9,40,41]. There is also the possibility that older adults are more aware of and accustomed to managing the symptoms of AUD or have learned to cope with them [42], potentially masking the severity of the disorder. Nevertheless, as the lack of significant age group differences in the likelihood of endorsing the loss of control, craving, tolerance, and withdrawal indicates, older adults who are addicted to alcohol are likely to engage in the same disordered alcohol consumption behaviors as younger age groups. With aging-related physiological changes, older adults are more vulnerable to the adverse consequences of AUD on physical, mental, and cognitive (including memory or attention) health, requiring increased medical attention [1].

### 4.2. CUD

Consistent with the bivariate analysis results, the multivariable analysis results show that the 65+ age group had lower odds of endorsing only two criteria (hazardous use and withdrawal). Understandably, older adults are less likely to engage in hazardous use of cannabis if they use it for a medical purpose, for example, for pain relief, sleep issues, or anxiety, which may not significantly interfere with their daily functioning or interpersonal relationships. Medical cannabis users may be more intentional about their cannabis use, perhaps using it in more controlled ways. As discussed, older adults with CUD may try to adopt safer and less harmful ways of use [34]. Since cannabis has long been stigmatized as an illicit drug, some older cannabis users tend to employ strategies to conceal their use or minimize stigma in accessing, using, and discussing their use with peers or healthcare providers [43,44,45]. The adoption of cautious strategies to avoid dealing with shame and stigma may also contribute to lowering withdrawal symptoms. A study found that cannabis users aged 50 and older reported fewer undesirable acute effects and withdrawal symptoms compared with younger users aged 18–29 [46].

Although it was hypothesized that the larger amount/longer duration, time spent, craving, and tolerance would be equally likely to be endorsed in both age groups, the lack of significant age group difference in social impairment criteria in CUD is intriguing. This null finding may be due to the small sample size for the 65+ age group and the small percentages of those who endorsed failure to fulfill role obligations and social problems in the under 65 age group, i.e., smaller than in the case of AUD. Cannabis, in some cases, might be perceived as less impairing. Thus, those with CUD might not perceive or report impairments in the same way they would with alcohol, particularly if cannabis is used for therapeutic or medicinal purposes. Recent studies showed that, in general, the risk perceptions of using cannabis 1–2 times a week among cannabis users were much lower than the risk of using alcohol 1–2 times a week [47,48]. Drug harm studies also showed that alcohol is the most harmful substance to self and society [49,50]. Cannabis, on the other hand, has shown some evidence for its therapeutic potential for neuropathic pain, seizure disorders, appetite stimulation, muscle spasticity, and treatment of cancer-related nausea/vomiting, thus creating a perception of cannabis having health-related utility [51,52,53]. However, it is notable that 80% of those with CUD in both age groups endorsed that they spent a great deal of time obtaining, using, or recovering, and close to 70% of the 65+ age group and 80% of the under 65 age group reported craving. These were significantly higher than among those with AUD and indicate the heavy toll of CUD in daily life.

### 4.3. Limitations

There were a few study limitations. First, the validity of respondents’ self-endorsement of SUD criteria was not checked by any supporting observation or evidence. Second, despite the three-year pooled data, the sample size for those with CUD in the 65+ age group was small. Third, given the small sample size, any subgroup analyses by gender and race/ethnicity were not attempted, which calls for further research in the future. Fourth, given that the COVID-19 pandemic was still ongoing in 2021 when people had fewer social demands, the findings need to be interpreted with caution.

### 4.4. Implications

Despite these limitations, the findings are informative, as they show the importance of better understanding the age-related social context in clinical assessment and treatment of AUD and CUD in older adults. Clinicians should be aware that older adults might not express the full range of SUD symptoms in the same way as younger individuals. Specific implications for clinical assessment and treatment engagement of older adults are as follows. First, for AUD, clinicians should consider that older adults may not report role or social impairment as a significant issue, even if alcohol is still causing harm in other ways. A more nuanced understanding of an older adult’s social context—such as their social network and role in family dynamics—may be needed when assessing AUD. This could include asking targeted questions about how alcohol use affects relationships or daily activities, beyond just assessing its impact on formal roles. Clinicians should also be aware that older adults might not connect their physical or psychological issues to their alcohol consumption, even if there is a clear relationship. Incorporating questions about how alcohol use affects physical, mental, and cognitive (including memory or attention) health, which also identifies potential hazards that older adults might overlook or underreport, can help identify subtle cases of AUD.

Second, when assessing CUD, clinicians should be aware that older adults might not perceive or report impairments in the same way they would with alcohol, particularly if cannabis is used for therapeutic or medicinal purposes. Even though older adults with CUD may not endorse or underreport physical or psychological problems in the same way they would with AUD, it is still crucial to assess for physical, psychological, and cognitive changes and the impact of these changes on their day-to-day functioning, overall quality of life, and potential long-term effects. Clinicians should be aware of the potential stigma surrounding cannabis use in older populations, which might prevent older adults from acknowledging its effects or seeking help, which could lead to a misinterpretation of the severity of their cannabis use and its impact on their functioning. The use of age-appropriate and substance-specific screening tools that take into account the unique ways in which cannabis might affect older adults, including its interaction with medications, health conditions, and lifestyle, would be helpful.

Third, more research is needed to expand our study to examine older adults who have other types of SUDs, such as opioid and other psychotherapeutic drug use disorders, illicit drug use disorders, poly-substance use disorders, e.g., AUD and CUD, and comorbid SUD and mental disorders. The findings from the expanded research are needed to enhance our understanding of the best ways to identify SUDs in older adults and help them better engage in treatment.

## 5. Conclusions

We found different age-related patterns of endorsing DSM-5 criteria for AUD and CUD, with more pronounced age group differences in AUD than in CUD. The significantly lower odds of older adults’ endorsement of seven AUD criteria point to the possibility that the overall prevalence of AUD, especially severe cases of AUD, may be underestimated in older adults. Our study also shows that regardless of age, those with CUD may be less inclined to perceive their CUD as causing significant harm, possibly because of societal shifts in cannabis perceptions and the legalization of its medical and recreational use over the recent decade. Despite the changes in the social and legal contexts, older adults with CUD might still carry stigmatized views about cannabis use in general, particularly if they grew up in an era when its use was heavily criminalized or frowned upon. This could lead to underreporting or denial of issues related to CUD, even if they are experiencing impairment. This study underscores the importance of better understanding the age-related social context in the clinical assessment and treatment of AUD and CUD in older adults.

## Figures and Tables

**Table 1:** Prevalence of alcohol and cannabis use and use disorder by age group among individuals aged 18 and older, 2021–2023 (%).

	Age Group						*p*
	All	18–25	26–34	35–49	50–64	65+	
% of All Age Groups	100	13.3	15.7	24.6	24.3	22.1	
Past-month alcohol use	52.0	50.0	60.3	56.1	51.9	43.1	<0.001
Past-year alcohol use	67.1	67.8	76.3	71.4	66.2	56.2	<0.001
Past-year alcohol use disorder	11.2	15.4	16.7	12.8	9.6	4.8	<0.001
Past-month cannabis use	15.4	25.2	24.1	16.6	12.2	5.7	<0.001
Past-year cannabis use	22.0	37.0	33.8	23.6	16.8	8.3	<0.001
Past-year cannabis use disorder	6.6	15.9	11.7	6.5	3.5	0.9	<0.001

Note: Respondents from the 2021–2023 National Survey on Drug Use and Health: Total sample *N* of all age groups (18–65+) = 139,524; total population *N* = 255,882,892. Probability values denote age group differences based on the omnibus Pearson’s χ^2^ tests.

**Table 2:** Characteristics of adults with alcohol and cannabis use disorders (% otherwise specified).

	Alcohol Use Disorder			Cannabis Use Disorder		
	65+	<65	*p*	65+	<65	*p*
*N* %	740 9.5	16,754 90.5		145 3.1	12,119 96.9	
Gender			0.067			0.076
Female	36.9	42.1		28.7	40.1	
Male	63.1	57.9		71.3	59.9	
Race/ethnicity			<0.001			0.264
Non-Hispanic White	80.0	62.6		72.4	59.4	
Non-Hispanic Black	8.5	12.2		9.4	15.2	
Hispanic	7.2	18.1		8.0	17.1	
Asian/Asian Pacific Islander	2.9	3.6		4.6	3.1	
American Indian/Alaska Native	0.6	0.7		2.1	1.1	
Multi-racial	0.8	2.8		3.5	4.1	
Education			0.954			0.483
≤High school	34.4	33.5		48.5	42.8	
Some college/associate degree	31.8	32.4		28.4	37.1	
College degree	33.8	34.1		23.1	20.1	
Work status			<0.001			<0.001
Employed full-time	12.0	60.5		8.7	49.1	
Employed part-time	15.5	13.4		9.2	16.4	
Unemployed	0.3	7.0		1.2	10.2	
Other	72.2	19.1		80.9	24.3	
Self-rated health (1 = poor, 5 = excellent), M (SE)	2.70 (0.06)	2.52 (0.01)	0.005	2.95 (0.14)	2.67 (0.02)	0.043

Probability values denote group differences based on Pearson’s χ^2^ or *t*-tests.

**Table 3:** Endorsement of *DSM-5* SUD criteria by age group (% otherwise specified).

Alcohol Use Disorder	Alcohol Use Disorder			Cannabis Use Disorder		
	65+	<65	*p*	65+	<65	*p*
*N* %	740 9.5	16,754 90.5		145 3.1	12,119 96.9	
Larger amount/longer duration	56.8	66.1	0.005	31.1	45.4	0.100
Unsuccessful efforts to cut down/control use	38.9	33.6	0.059	18.1	26.1	0.116
A great deal of time is spent obtaining, using, and/or recovering	39.9	55.0	<0.001	79.4	81.0	0.734
Craving/strong urge to use	66.4	69.1	0.296	68.5	78.8	0.070
Recurrent use failing to fulfill major role obligations at work, school, or home	3.3	8.6	0.001	3.6	6.6	0.457
Continued use despite social problems	9.3	16.8	<0.001	4.9	6.3	0.705
Important social, occupational, or recreational activities given up or reduced	11.7	15.1	0.093	7.7	14.0	0.195
Recurrent use in physically hazardous situations	8.7	13.2	0.023	0.2	3.5	<0.001
Continued use despite physical or psychological problems	19.0	31.0	<0.001	12.1	11.9	0.975
Tolerance	45.3	51.1	0.109	50.6	59.0	0.257
Withdrawal	43.0	45.2	0.550	41.6	65.6	0.003
Withdrawal symptoms	42.7	44.4	0.663	41.5	64.9	0.004
The same/related substance taken to avoid withdrawal	8.1	10.1	0.325	2.0	11.4	<0.001
No. of endorsed criteria, M (SE)	3.42 (0.11)	4.05 (0.03)	<0.001	3.18 (0.15)	3.98 (0.03)	<0.001
Symptom severity			<0.001			0.012
Mild	69.0	53.8		70.6	0.100	
Moderate	16.4	24.3		25.4	0.116	
Severe	14.6	22.0		4.0	0.734	

Note: Probability values denote group differences based on Pearson’s χ^2^ or *t*-tests.

**Table 4:** Associations between age group and each criterion endorsement for AUD and CUD: logistic regression results.

	Alcohol Use Disorder		Cannabis Use Disorder	
	aOR (95% CI)		aOR (95% CI)	
	65+ vs. <65 Years	Model Statistics	65+ vs. <65 Years	Model Statistics
Larger amount/longer duration	0.71 (0.52–0.96) *	F(13,38) = 4.93; *p* < 0.001	0.54 (0.27–1.10)	F(13,38) = 5.68; *p* < 0.001
Loss of control	1.21 (0.92–1.58)	F(13,38) = 3.91; *p* < 0.001	0.70 (0.39–1.23)	F(13,38) = 3.26; *p* = 0.002
A great deal of time	0.59 (0.44–0.78) **	F(13,38) = 6.73; *p* < 0.001	1.15 (0.66–2.01)	F(13,38) = 3.66; *p* < 0.001
Craving	0.76 (0.56–1.02)	F(13,38) = 8.04; *p* < 0.001	0.61 (0.34–1.12)	F(13,38) = 3.53; *p* = 0.001
Role fulfillment	0.30 (0.16–0.56) ***	F(13,38) = 23.00; *p* < 0.001	0.52 (0.09–2.98)	F(13,38) = 7.12; *p* < 0.001
Social problems	0.46 (0.30–0.71) **	F(13,38) = 12.58; *p* < 0.001	0.79 (0.18–3.55)	F(13,38) = 4.38; *p* < 0.001
Activities	0.66 (0.47–0.94) *	F(13,38) = 15.60; *p* < 0.001	0.57 (0.20–1.67)	F(13,38) = 3.76; *p* < 0.001
Hazardous situations	0.53 (0.34–0.81) **	F(13,38) = 12.09; *p* < 0.001	0.04 (0.01–0.17) ***	F(13,38) = 5.35 *p* < 0.001
Physical/psychological problems	0.51 (0.37–0.70) ***	F(13,38) = 13.39; *p* < 0.001	0.98 (0.36–2.63)	F(13,38) = 5.52; *p* < 0.001
Tolerance	0.79 (0.58–1.09)	F(13,38) = 6.78; *p* < 0.001	0.74 (0.41–1.34)	F(13,38) = 1.47; *p* = 0.173
Withdrawal A	0.78 (0.58–1.04)	F(13,38) = 16.29; *p* < 0.001	0.39 (0.20–0.73) **	F(13,38) = 5.44; *p* < 0.001
Withdrawal B	0.69 (0.39–1.21)	F(13,38) = 8.28; *p* < 0.001	0.16 (0.05–0.48) **	F(13,38) = 3.49; *p* = 0.001
*N* for each model	17,489 (population size = 28,203,184)		12,263 (population size = 16,523,201)	
Design *df*	50		50	

Covariates controlled in each model: Gender, race/ethnicity, education, work status, and self-rated health. * *p* < 0.05; ** *p* < 0.01; *** *p* < 0.001.

## Data Availability

This study is based on de-identified public-domain data (The National Survey on Drug Use and Health).

## References

[1] Barry K.L., Blow F.C. (2016). Drinking over the lifespan: Focus on older adults. Alcohol Res. Curr. Rev..

[2] Kuerbis A. (2020). Substance use among older adults: An update on prevalence, etiology, assessment, and intervention. Gerontology.

[3] Hu J., Kulkarni N., Maliha P., Grossberg G. (2024). Prevalence and treatment of substance misuse in older adults: Beyond early adulthood. Subst. Abuse Rehabil..

[4] Keyes K.M. (2023). Alcohol use in the older adult US population: Trends, causes, and consequences. Alcohol.

[5] Lin J., Arnovitz M., Kotbi N., Francois D. (2023). Substance use disorders in the geriatric population: A review and synthesis of the literature of a growing problem in a growing population. Curr. Treat. Options Psychiatry.

[6] Waddell J.T. (2022). Age-varying time trends in cannabis- and alcohol-related risk perceptions 2002–2019. Addict. Behav..

[7] Dowling G.J., Weiss S.R., Condon T.P. (2008). Drugs of abuse and the aging brain. Neuropsychopharmacology.

[8] Fagiolino P., Eiraldi R., Vázquez M. (2006). The influence of cardiovascular physiology on dose/pharmacokinetic and pharmacokinetic/pharmacodynamic relationships. Clin. Pharmacokinet..

[9] Fenollal-Maldonado G., Brown D., Hoffman H., Kahlon C., Grossberg G. (2022). Alcohol use disorder in older adults. Clin. Geriatr. Med..

[10] Schröder S., Westhoff M.S., Pfister T., Seifert J., Bleich S., Koop F., Proskynitopoulos P.J., Glahn A., Heck J. (2024). Drug safety in older patients with alcohol use disorder: A retrospective cohort study. Ther. Adv. Psychopharmacol..

[11] Chhatre S., Cook R., Mallik E., Jayadevappa R. (2017). Trends in substance use admissions among older adults. BMC Health Serv. Res..

[12] Choi N.G., DiNitto D.M., Marti C.N., Choi B.Y. (2022). U.S. older adults’ heroin and psychostimulant use treatment admissions, 2012–2019: Sociodemographic and clinical characteristics. Drug Alcohol Depend..

[13] Lynch A., Arndt S., Acion L. (2021). Late- and typical-onset heroin use among older adults seeking treatment for opioid use disorder. Am. J. Geriatr. Psychiatry.

[14] Na P.J., Rosenheck R., Rhee T.G. (2022). Increased admissions of older adults to substance use treatment facilities and associated changes in admission characteristics, 2000–2017. J. Clin. Psychiatry.

[15] Choi N.G., DiNitto D.M. (2019). Older marijuana users in substance abuse treatment: Treatment settings for marijuana-only versus polysubstance use admissions. J. Subst. Abuse Treat..

[16] Choi N.G., Moore J., Choi B.Y. (2024). Cannabis use disorder and substance use treatment among U.S. adults. J. Subst. Use Addict. Treat..

[17] Han B.H., Palamar J.J. (2018). Marijuana use by middle-aged and older adults in the United States, 2015–2016. Drug Alcohol Depend..

[18] Choi N.G., Marti C.N. (2025). Treatment use among U.S. adults with a substance use disorder: Associations with symptom severity, problem self-perception, comorbid mental illness, and mental health treatment. Int. J. Environ. Res. Public Health.

[19] American Psychiatric Association (2013). Diagnostic and Statistical Manual of Mental Disorders.

[20] Kuerbis A., Sacco P., Blazer D.G., Moore A.A. (2014). Substance abuse among older adults. Clin. Geriatr. Med..

[21] Bailey A.J., Quinn P.D., McHugh R.K. (2025). Implications of the shift to DSM-5 for alcohol use disorder prevalence estimates in the National Survey on Drug Use and Health. Addiction.

[22] Compton W.M., Han B., Jones C.M., Blanco C. (2019). Cannabis use disorders among adults in the United States during a time of increasing use of cannabis. Drug Alcohol Depend..

[23] Caulkins J.P. (2024). Changes in self-reported cannabis use in the United States from 1979 to 2022. Addiction.

[24] Choi N.G., DiNitto D.M. (2021). Comparing older nonmedical and medical cannabis users: Health-related characteristics, cannabis use patterns, and cannabis sources. Am. J. Drug Alcohol Abuse.

[25] Fink D.S., Shmulewitz D., Mannes Z.L., Stohl M., Livne O., Wall M., Hasin D.S. (2022). Construct validity of DSM-5 cannabis use disorder diagnosis and severity levels in adults with problematic substance use. J. Psychiatr. Res..

[26] Hallgren K.A., Matson T.E., Oliver M., Witkiewitz K., Bobb J.F., Lee A.K., Caldeiro R.M., Kivlahan D., Bradley K.A. (2022). Practical assessment of alcohol use disorder in routine primary care: Performance of an alcohol symptom checklist. J. Gen. Intern. Med..

[27] Kervran C., Shmulewitz D., Serre F., Denis C., Roux P., Jauffret-Roustide M., Lalanne L., Hasin D., Auriacombe M. (2021). Do DSM-5 substance use disorder criteria differ by user care settings? An item response theory analysis approach. Addict. Behav..

[28] Mannes Z.L., Shmulewitz D., Livne O., Stohl M., Hasin D.S. (2021). Correlates of mild, moderate, and severe alcohol use disorder among adults with problem substance use: Validity implications for DSM-5. Alcohol. Clin. Exp. Res..

[29] Li L., Deng Q.C. (2024). Loneliness and cannabis use among older adults: Findings from a Canada national survey during the COVID-19 pandemic. BMC Public Health.

[30] Preuss U.W., Watzke S., Wurst F.M. (2014). WHO/ISBRA Study on State, & Trait Markers of Alcohol Use and Dependence Investigators. Dimensionality and stages of severity of DSM-5 criteria in an international sample of alcohol-consuming individuals. Psychol. Med..

[31] Pringle K.E., Heller D.A., Ahern F.M., Gold C.H., Brown T.V. (2006). The role of medication use and health on the decision to quit drinking among older adults. J. Aging Health.

[32] Dawson D.A., Goldstein R.B., Grant B.F. (2013). Prospective correlates of drinking cessation: Variation across the life-course. Addiction.

[33] Gavens L., Goyder E., Hock E.S., Harris J., Meier P.S. (2016). Alcohol consumption after health deterioration in older adults: A mixed-methods study. Public Health.

[34] Lau N., Sales P., Averill S., Murphy F., Sato S.O., Murphy S. (2015). Responsible and controlled use: Older cannabis users and harm reduction. Int. J. Drug Policy.

[35] Center for Behavioral Health Statistics and Quality [CBHSQ] (2024). 2023 National Survey on Drug Use and Health Public Use File Codebook.

[36] Center for Behavioral Health Statistics and Quality [CBHSQ] (2023). 2022 National Survey on Drug Use and Health Public Use File Codebook.

[37] Allison P. (2012). How Can You Safely Ignore Multicollinearity?. https://statisticalhorizons.com/multicollinearity/.

[38] Kuerbis A.N., Hagman B.T., Sacco P. (2013). Functioning of alcohol use disorders criteria among middle-aged and older adults: Implications for DSM-5. Subst. Use Misuse.

[39] DiBartolo M.C., Jarosinski J.M. (2017). Alcohol use disorder in older adults: Challenges in assessment and treatment. Issues Ment. Health Nurs..

[40] Le Roux C., Tang Y., Drexler K. (2016). Alcohol and opioid use disorder in older adults: Neglected and treatable illnesses. Curr. Psychiatry Rep..

[41] Loscalzo E., Sterling R.C., Weinstein S.P., Salzman B. (2017). Alcohol and other drug use in older adults: Results from a community needs assessment. Aging Clin. Exp. Res..

[42] Substance Abuse and Mental Health Services Administration Treating Substance Use Disorder in Older Adults, Updated 2020: Treatment Improvement Protocol, TIP 26. Substance Abuse and Mental Health Services Administration 2020, PEP20-02-01-011. https://library.samhsa.gov/sites/default/files/tip-26-pep20-02-01-011.pdf.

[43] Bobitt J., Qualls S.H., Schuchman M., Wickersham R., Lum H.D., Arora K., Milavetz G., Kaskie B. (2019). Qualitative analysis of cannabis use among older adults in Colorado. Drugs Aging.

[44] Dahlke S., Butler J.I., Hunter K.F., Toubiana M., Kalogirou M.R., Shrestha S., Devkota R., Law J., Scheuerman M. (2024). The effects of stigma: Older persons and medicinal cannabis. Qual. Health Res..

[45] Abu Baker D., Cruz Rivera P.N., Narasimhan R., Nguyen N., Tibiriçá L., Kepner W.E., O’Malley P., Nguyen A.L., Moore A.A. (2024). Older adults’ use of cannabis and attitudes around disclosing medical cannabis use to their healthcare providers in California: A mixed methods study. Drugs Real World Outcomes.

[46] Sexton M., Cuttler C., Mischley L.K. (2019). A survey of cannabis acute effects and withdrawal symptoms: Differential responses across user types and age. J. Altern. Complement. Med..

[47] Choi N.G., Marti C.N., Choi B.Y. (2024). Perceived risk of binge drinking among older alcohol users: Associations with alcohol use frequency, binge drinking, alcohol use disorder, and alcohol treatment use. Int. J. Environ. Res. Public Health.

[48] Choi N.G., Marti C.N., Choi B.Y. (2024). Associations between cannabis consumption methods and cannabis risk perception. Int. J. Environ. Res. Public Health.

[49] Bonomo Y., Norman A., Biondo S., Bruno R., Daglish M., Dawe S., Egerton-Warburton D., Karro J., Kim C., Lenton S. (2019). The Australian drug harms ranking study. J. Psychopharmacol..

[50] Crossin R., Cleland L., Wilkins C., Rychert M., Adamson S., Potiki T., Pomerleau A.C., MacDonald B., Faletanoai D., Hutton F. (2023). The New Zealand drug harms ranking study: A multi-criteria decision analysis. J. Psychopharmacol..

[51] Doppen M., Kung S., Maijers I., John M., Dunphy H., Townsley H., Eathorne A., Semprini A., Braithwaite I. (2022). Cannabis in palliative care: A systematic review of current evidence. J. Pain Symptom Manag..

[52] Jeddi H.M., Busse J.W., Sadeghirad B., Levine M., Zoratti M.J., Wang L., Noori A., Couban R.J., Tarride J.E. (2024). Cannabis for medical use versus opioids for chronic non-cancer pain: A systematic review and network meta-analysis of randomised clinical trials. BMJ Open.

[53] Senderovich H., Patel P., Jimenez Lopez B., Waicus S. (2022). A systematic review on cannabis hyperemesis syndrome and its management options. Med. Princ. Pract..

